# Shifting the pH Optima of (*R*)-Selective Transaminases by Protein Engineering

**DOI:** 10.3390/ijms232315347

**Published:** 2022-12-05

**Authors:** Chao Xiang, Yu-Fei Ao, Matthias Höhne, Uwe T. Bornscheuer

**Affiliations:** 1 Department of Biotechnology and Enzyme Catalysis, Institute of Biochemistry, University of Greifswald, 17487 Greifswald, Germany; 2 Beijing National Laboratory for Molecular Sciences, CAS Key Laboratory of Molecular Recognition and Function, Institute of Chemistry, Chinese Academy of Sciences, Beijing 100190, China; 3 University of Chinese Academy of Sciences, Beijing 100049, China

**Keywords:** amine transaminases, asymmetric synthesis, pH optimum, protein engineering, rational design

## Abstract

Amine transaminases (ATAs) are powerful biocatalysts for the stereoselective synthesis of chiral amines. However, wild-type ATAs usually show pH optima at slightly alkaline values and exhibit low catalytic activity under physiological conditions. For efficient asymmetric synthesis ATAs are commonly used in combination with lactate dehydrogenase (LDH, optimal pH: 7.5) and glucose dehydrogenase (GDH, optimal pH: 7.75) to shift the equilibrium towards the synthesis of the target chiral amine and hence their pH optima should fit to each other. Based on a protein structure alignment, variants of (*R*)-selective transaminases were rationally designed, produced in *E. coli*, purified and subjected to biochemical characterization. This resulted in the discovery of the variant E49Q of the ATA from *Aspergillus fumigatus*, for which the pH optimum was successfully shifted from pH 8.5 to 7.5 and this variant furthermore had a two times higher specific activity than the wild-type protein at pH 7.5. A possible mechanism for this shift of the optimal pH is proposed. Asymmetric synthesis of (*R*)-1-phenylethylamine from acetophenone in combination with LDH and GDH confirmed that the variant E49Q shows superior performance at pH 7.5 compared to the wild-type enzyme.

## 1. Introduction

The chiral amine group occurs as the core structure in numerous natural products and synthetic pharmaceuticals and chemicals. Enantiopure amines are frequently used as key chiral building blocks for these bioactive drugs and agrochemicals, and therefore have attracted particular attention for synthetic chemists [1]. Many chemical synthetic strategies have been developed to access chiral amines, including traditional resolution processes using chiral acids or asymmetric synthesis using transition metals or organic small molecule catalysts, but these methods suffer from the use of stoichiometric chiral resolution reagents or toxic components [2,3]. On the other hand, various enzymes, including hydrolases, oxidoreductases and transferases, can be employed to make chiral amines using environmentally friendly and highly selective methods [4,5,6]. This includes transaminases, pyridoxal-5′-phosphate (PLP)-dependent enzymes that catalyze the asymmetric amination of a ketone to the corresponding amine. These biocatalysts exhibit high enantioselectivity and broad substrate tolerance and are thus widely applied for the preparation of optically pure amines [7,8,9,10]. The most popular example is the transaminase-catalytic asymmetric synthesis of the antidiabetic drug (*R*)-sitagliptin, with >99.95% optical purity developed by researchers from Merck & Co. Inc. (Rahway, NJ, USA) and Codexis, Inc. (Redwood City, CA, USA), which replaced the previously developed asymmetric chemical hydrogenation [11]. Based on their enantiopreference, transaminases are classified into two types, (*S*)- and (*R*)-selective enzymes, which belong to the fold types I and IV, respectively, and they differ substantially in their protein structure [7,8,9,10].

Transaminases usually show highest specific activity under slightly basic conditions, as reported by us for seven (*R*)-selective transaminases [12,13,14]. For efficient asymmetric synthesis, the equilibrium must be shifted towards product synthesis and commonly lactate dehydrogenase (LDH, optimal pH: 7.5) and glucose dehydrogenase (GDH, optimal pH: 7.75) are used together with the TA to convert the pyruvate formed from the amine donor (D- or L-Ala) into lactic acid [15]. Furthermore, transaminases are also used in enzyme cascade reactions, for instance in combination with enoate reductases [16] or ketoreductases [17,18], to make chiral products. This prompted us to use methods of protein engineering to shift the pH optimum of TAs towards neutral pH values.

Protein engineering is powerful in improving various enzymatic catalytic features such as substrate promiscuity, catalytic activity and selectivity, enzymatic stability and pH optimum [19,20,21]. Generally, the most universal and effective method of modifying pH optimum is to change the electrostatic environment of the active site by protein engineering to alter the p*K*a values of amino acid side chains in the protein [22,23,24,25,26,27,28,29,30,31,32,33,34,35,36,37]. So far, a series of enzymes such as serine proteases [22,23,24], xylanases [25,26,27,28], phytases [29,30], DNase I [31], phospholipase [32] or an aspartase [33], have been successfully engineered for optimal pH values through modifying their surface charge or mutating crucial residues adjacent to their active site. Most of these enzymes have a Ser or Glu residue in the active site and follow a general acid-base catalytic mechanism. However, the mechanisms of PLP-dependent enzymes are quite different, and few reports have exploited alteration of their pH optima [38].

For transaminases, the Kagamiyama group [39,40,41,42] has explored the catalytic mechanism of an aspartate aminotransferase and found that the imine-pyridine torsion of the Schiff base intermediate, other than electrostatic interaction, is crucial for the low p*K*a value of the aldimine group of the PLP-cofactor, which results in the amino-transfer reaction under neutral or slightly alkaline condition but without a focus on pH optimum. As far as we know, the only literature report is from the Berglund group [43], who found that the (*S*)-selective wild-type *Chromobacterium violaceum* ATA has an optimum pH at 8.3, but its Trp60Cys variant showed an optimum at pH 7.0. They offered the possible explanation that the introduced cysteine residue participates in the reaction and affects the binding of the cofactor to the enzyme.

In contrast, although many reports have been published about the improvement of catalytic activity, substrate scope and selectivity of (*R*)-selective transaminase by protein engineering [44], pH optimum adjustments remained unexplored. We have thus aimed to alter the pH optimum of (*R*)-TAs using rational protein design for the creation of biocatalysts active around neutral pH-values.

## 2. Results and Discussion

**Figure 1:**
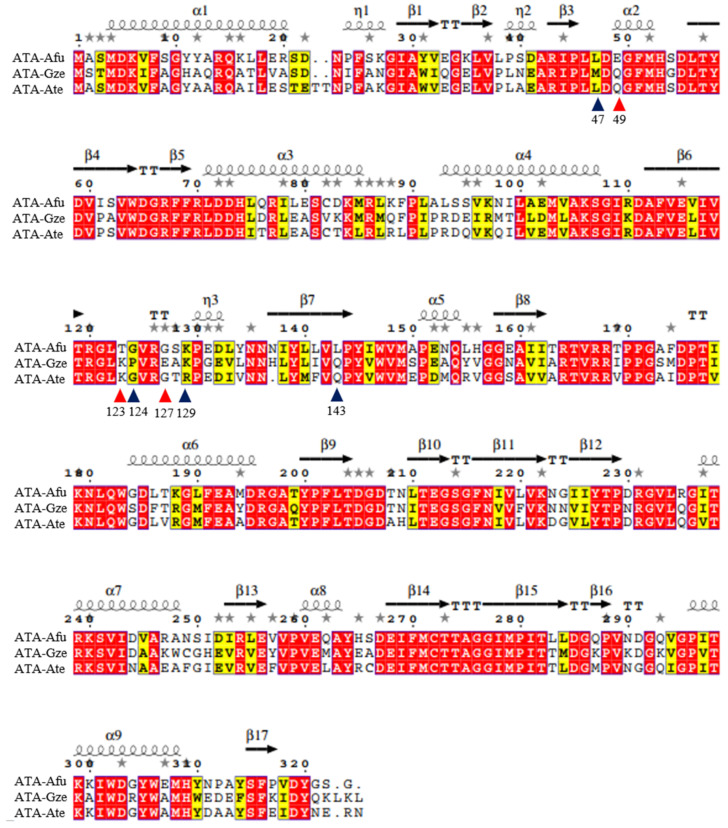
Amino acid sequence alignments of ATA-Afu, ATA-Gze and ATA-Ate. The residues in the substrates tunnel area are marked with triangles. Among them, E49, T123 and G127 present in ATA-Afu (red colored triangles) have differently charged side chains compared to the other two ATAs. The red box and white character mean that the residues are identical. The yellow box and black character mean that the residues are similar in size and charge. The figure was created using the online alignment tools *Cobalt* [47] and *ESPript 3.0* [48].

**Figure 2:**
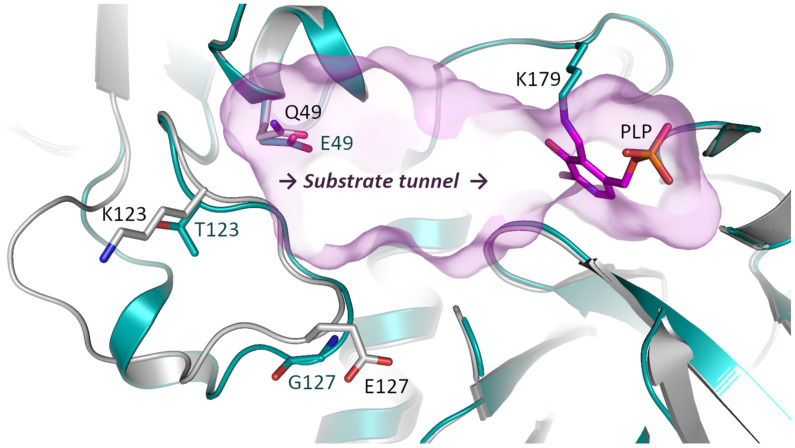
Targeted residues in the substrates tunnel area of ATA-Afu and ATA-Gze. The proteins ATA-Afu and ATA-Gze are shown as cyan and white cartoons, respectively. The oxygen and nitrogen atoms of different residues are colored red and blue. The carbon atoms of the cofactor PLP are colored purple. The substrate tunnel is colored light purple. The figure was created using *PyMOL 2.3.0* [49].

In our previous study we have found several (*R*)-selective transaminases and investigated their pH-activity profiles [12,13]. Interestingly, three of these highly homologous transaminases (ATA-Afu from *Aspergillus fumigatus*, ATA-Gze from *Gibberella zeae* and ATA-Ate from *Aspergillus terreus*) showed different pH profiles. ATA-Afu possessed an optimum at pH 8.5 while ATA-Gze or ATA-Ate showed an optimum at pH 7.5. We compared the 3D structures of ATA-Afu (PDB: 4CHI), ATA-Gze (homology modeling by *YASARA 17.1.28* [45]) and ATA-Ate (PDB: 4CE5), but all the residues in the catalytic cavity are conserved. Therefore, we expanded our search to the entire tunnel area and found surface residues that differ in their charge, which may affect the surface electrostatic environment (Figure 1 and Figure 2). As shown in Figure 2, the residues E49, T123 and G127 present in ATA-Afu have different charges and sizes compared to Q49, K123 and E127 present in ATA-Gze. An evolutionary conservation analysis [46] also indicates that these residues are not conserved (Figure S6). We thus expected that an exchange of these key residues may alter the pH optimum of ATA-Afu. According to this hypothesis, we created the variants encoding for the substitutions E49Q, T123K and G127E of ATA-Afu, Q49E, K123T and E127G of ATA-Gze and Q51E, K125T and G129E of ATA-Ate to investigate how these positions affect the pH optimum in general. All variants were functionally expressed in *E. coli*, purified and subjected to biochemical characterization with a special focus on their pH optima.

Activity tests were performed using Davies buffer [50] at pH values ranging from 6.0 to 9.5 using the photometric acetophenone assay in the transaminase-catalyzed reaction between (*R*)-1-phenylethylamine and pyruvate [51].

**Figure 3:**
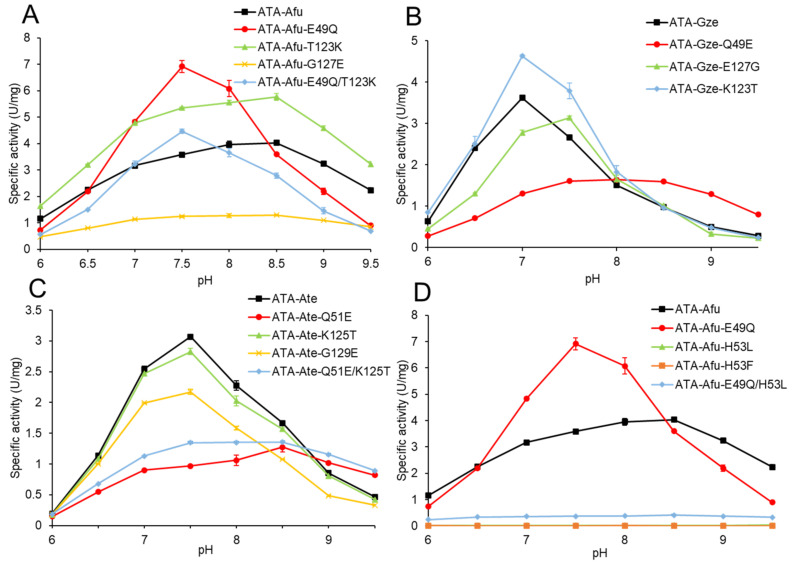
pH activity profiles of wild-type and variants of ATA-Afu (**A**,**D**), ATA-Gze (**B**) and ATA-Ate (**C**). The pH optimum was determined by assessing the specific activities using the acetophenone assay at different pH conditions. Data points correspond to the mean values of three independent experiments. The error bars are shown in the figure and the detailed data for specific activity measurements are given in Table S2. The kinetic parameters are shown in Table S3 and Figure S1.

As shown in Figure 3A, wild-type ATA-Afu exhibited its maximum activity at pH 8.5. To our delight, replacing the acidic residue E49 with the neutral residue Q lowered the pH optimum by 1.0 unit to pH 7.5. Moreover, the specific activity of the ATA-Afu-E49Q variant was two times higher than for the wild-type at pH 7.5. When the neutral residue T123 of this enzyme was replaced by the basic residue K, no change in the optimal pH was observed, but the specific activity was 1.5-fold higher at pH 7.5. On the contrary, variant G127E showed a flat pH profile and the specific activity is lower than for the other variants. A combination of the substitutions E49Q and T123K resulted in a pH optimum of 7.5, but its specific activity was slightly lower than that determined for the variant E49Q.

Both wild-type ATA-Gze and ATA-Ate exhibit their maximum activity at pH 7.5 (Figure 3B,C). When their neutral residues Q49 or Q51 were replaced by the acidic residue E, the pH optima were shifted to 8.0 or 8.5, respectively, and pH-activity profiles were similar to wild-type ATA-Afu. Substitution of residue K123/125 in ATA-Gze/ATA-Ate to threonine did not show a shift of their pH optima, but ATA-Gze-K123T displayed slightly increased specific activity at the optimal pH, consistent with the experimental results found for ATA-Afu (variant E49Q and E49Q/T123K). The substitutions E127G in ATA-Gze and G129E in ATA-Ate also showed the same pH optima but lower specific activities. The double variant ATA-Ate-Q51E/K125T showed a rather flat pH profile and its maximum value appeared at 8.5; its activity was slightly higher than found for variant ATA-Ate-Q51E. In summary, the trend of the pH-optima shifting observed for ATA-Gze or the ATA-Ate variants was consistent with the results found for the ATA-Afu variants.

**Figure 4:**
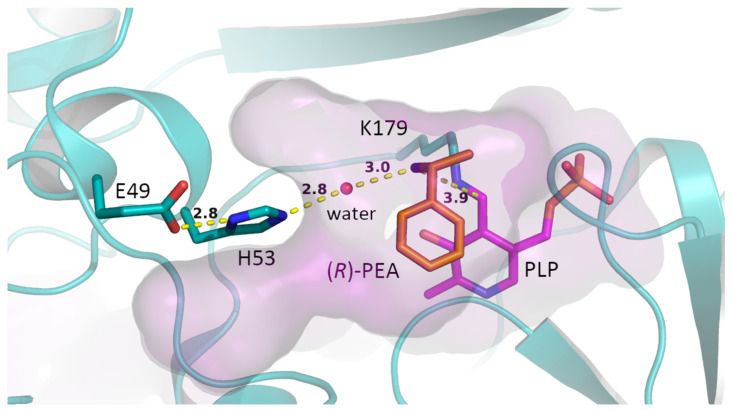
Substrate (*R*)-PEA accommodated in the active site of ATA-Afu. The protein is shown in cyan. The tunnel is colored light purple. The oxygen and nitrogen atoms of residues and substrates are colored red and blue, respectively. The carbon atoms of the substrate are colored orange. Distances are represented by yellow dashed lines. The figure was created using PyMOL [49]. The position of the water molecule was assumed from the crystal structure of ATA-Afu (PDB: 4CHI). The superposed structure of the substrate is shown in Figure S5.

To study the origins of the shifted pH optima we performed molecular docking of the substrate (*R*)-1-phenylethylamine (*R*-PEA) into the catalytic cavity of ATA-Afu (Figure 4). To initiate the first half reaction of the catalytic cycle, the substrate’s amino group must be deprotonated so that it can act as nucleophile and attack the cofactor PLP (in its internal aldimine form). We speculated that the amino acid substitutions affect the enzyme’s ability: (i) to bind the substrate (*R*)-PEA in its deprotonated form; or (ii) to deprotonate (*R*)-PEA so that the catalytic cycle can be initiated. Interestingly, residue E49 is rather far away from either the amino group of the substrate (11 Å) or the PLP cofactor (14 Å). This long distance means either further residues in the vicinity or water molecules have to mediate the effect of the E49 substitution onto the cofactor/(*R*)-PEA interaction. As shown in Figure 4, H53, which is located between E49 and the amino group of the substrate, probably acts as a base and deprotonates the incoming (*R*)-PEA, and the p*K*a of H53′s imidazole side chain might increase by the interaction between E49 and H53. Furthermore, the variants ATA-Afu-H53L and H53F were constructed, purified, and their activities measured in Davies buffers (pH range from 6.0 to 9.5). Both variants lost virtually all activity (Figure 3D). Considering the similar sterical hindrance but different electronic properties between residues H and L/F, we prefer to attribute the reduced activity to the role of H53 as a base, although we cannot exclude that this is due to a less accessible active site or non-competent binding of the substrate. Moreover, the double variant ATA-Afu-H53L/E49Q also exhibited extremely low specific activity. For ATA-Gze and ATA-Ate, variants H53L and H55L, respectively, showed similar pH profiles as ATA-Afu-H53L/E49Q (Figure S2, Supplementary Materials). Due to the low activities observed for the above three variants, their pH profiles are difficult to interpret.

**Figure 5:**
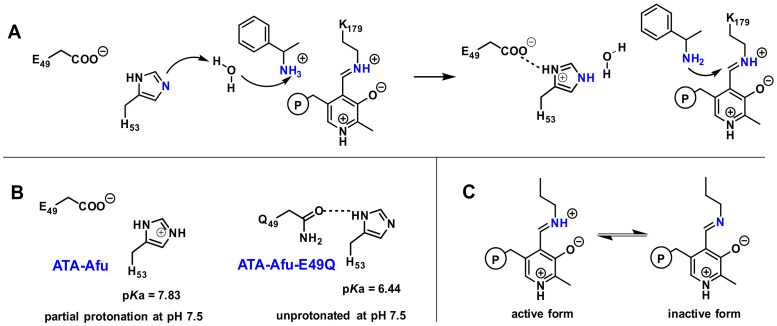
Postulated mechanism for the altered pH optimum of the E49Q variant of ATA-Afu. (**A**) H53 and E49 mediate the deprotonation of the substrate’s amino group. (**B**) The p*K*a value of the imidazole group of residue H53 is lowered by the E49Q substitution. (**C**) Internal aldimine of the PLP-cofactor. The negative charge is presumably stabilized by the neighboring residue Y58.

To further study the function of residue H55, we used the program *PropKa 3.3* [52], which calculated a predicted p*Ka* value of 7.83 for the H53 side chain of wild-type ATA-Afu. When substituting the neighboring E49 residue by a glutamine (E49Q), *PropKa* predicted a significantly lowered p*K*a value of 6.44 for the H53 imidazole group. According to this analysis, we propose the following hypothesis (Figure 5): (1) the E49Q substitution destabilizes the positively charged form of the imidazole group of H53. As this residue is partially exposed to water molecules and via the substrate entrance tunnel in contact with the bulk solvent, pH changes of the reaction medium will affect its protonation state in the following way: In the wild-type ATA-Afu, H53′s imidazole group is mainly in its protonated form if the pH value is below 7.83. This explains the lower activity at pH 7.5 than at 8.5. 

As the predicted p*K*a of residue H53 in ATA-Afu-E49Q is 6.44 it is mainly (>90%) deprotonated at pH 7.5, corresponding to the lower pH optimum compared to the wild type and a higher activity at pH 7.5; (2) The cofactor PLP forms a Schiff base with the ε-amino group of residue K179—the so-called internal aldimine. This internal aldimine exhibits a p*K*a value of 9.0 for ATA-Afu-E49Q, similar to the reported p*Ka* value (8.8) for the internal aldimine of an aspartate aminotransferase [42]. When the iminium ion is deprotonated under basic conditions, the resulting imine group shows a weaker electrophilicity, which corresponds to a lower catalytic activity. This is in line with the rapid decrease in catalytic activity at more basic conditions. It is worth noting that the pH profile of transaminases is influenced by all dissociable groups in the enzyme, and the mechanism of transaminase-catalysis is also very complex [53]. Although it is hard to provide a clear explanation for the shift of the pH optima, the pKa value of E49 indeed shows a positive influence on the pH profile and the above proposed mechanism provides a reasonable explanation for the experimental results.

**Figure 6:**
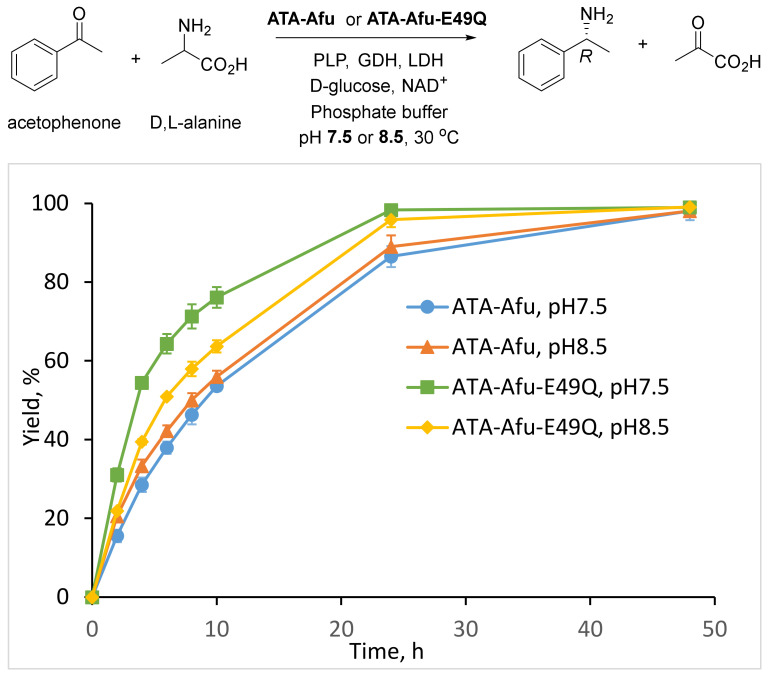
Asymmetric synthesis of (*R*)-PEA from acetophenone. Assay conditions for asymmetric synthesis: 10 mL final volume, 50 mM phosphate buffer at pH 7.5 or 8.5, 0.1 mM PLP, 5 mM ketone, 100 mM D,L-alanine, NAD^+^ (1 mM), GDH (1 U mL^−1^), D-glucose (50 mM) and LDH (3 U mL^−1^), DMSO 5%, 5 mg purified enzyme, 30 °C under stirring.

To demonstrate the synthetic applicability of the optimized variant ATA-Afu-E49Q we performed the asymmetric synthesis of (*R*)-PEA at pH 7.5 and 8.5 using the wild type transaminase as a control (Figure 6). To shift the equilibrium, the pyruvate removal system comprising LDH and GDH [14] was applied and the reaction progress was monitored by HPLC (Table S4). As already observed in the acetophenone assay experiments, the E49Q variant exhibited two times higher reaction rates compared to the wild-type at pH 7.5 and nearly 1.5-fold higher rates than the wild-type at pH 8.5. As reported in literature [54], the pH optima of LDH and GDH are 7.5 and 7.75, respectively, in line with the pH optimum of the newly designed ATA-Afu-E49Q variant, which explains its superior performance at pH 7.5.

## 3. Materials and Methods

### 3.1. Site-Directed Mutagenesis

All variants were prepared using the Q5^®^ site-directed mutagenesis kit from New England BioLabs. Degenerate primers were designed non-overlapping by using the standard setting of the NEBaseChanger. For the PCR, 0.25 ng μL^−1^ template plasmid (carrying the ATA-Afu, ATA-Gze, and ATA-Ate gene), 0.5 μM forward and reverse primers (Table S1), Q5^®^ hot start high-fidelity 2× master mix were used. The PCR was performed as follows: (i) 98 °C, 30 s; (ii) 30 cycles: 98 °C, 10 s; 50–72 °C, 30 s; 72 °C, 0.5 min/kbp; (iii) 72 °C, 2 min. The resulting PCR product was directly treated with the kinase, ligase and *Dpn*I (KLD enzyme mix) (100 μL mL^−1^; NEB) at room temperature for 30 min and then used for the transformation of chemically competent *E. coli* TOP10 cells. After confirming the introduced mutation by single colonies sequence detection, the aimed plasmids were used in the transformation of chemical competent *E. coli* BL21(DE3) cells by the heat shock method.

### 3.2. Gene Expression and Purification of the Enzyme Variants

For the protein biosynthesis of the ATA-Afu, ATA-Gze, and ATA-Ate and variants, transformed *E. coli* BL21(DE3) cells were incubated overnight at 37 °C in a 5 mL LB-medium (Lysogeny Broth) preculture with ampicillin (100 µg mL^−1^). An amount of 1 mL of the preculture was used for the inoculation of 100 mL TB-medium (supplemented with the corresponding antibiotic) and incubated at 37 °C, 180 rpm. The expression of the ATA-Afu, ATA-Gze, and ATA-Ate variants was induced at an optical density of approx. 0.6 (measured at 600 nm) with 0.2% L-rhamnose and incubated at 20 °C for 20 h. The cells were harvested by centrifugation (20 min, 4000× *g*, 4 °C).

For purification, the harvested cells were resuspended in 50 mM HEPES buffer pH 7.5 containing 0.1 mM PLP, 0.3 M NaCl, 0.01 M imidazole and lysed via ultrasound (50% pulse, 50% power, 2 × 5 min; Sonoplus HD2070, Bandelin Electronic GmbH, Berlin, Germany). The lysate was clarified by centrifugation (0.5 h, 10,000× *g*, 4 °C) and purified by immobilized metal affinity chromatography with the following buffers: washing buffer (50 mM HEPES buffer pH 7.5 containing 0.1 mM PLP, 0.3 M NaCl, 0.02 M imidazole), elution buffer (50 mM HEPES buffer pH 7.5 containing 0.1 mM PLP, 0.3 M NaCl, 0.3 M imidazole). The proteins were desalted in 50 mM HEPES buffer pH 7.5, 0.1 mM PLP using PD-10 desalting columns (GE Healthcare). The purified and desalted proteins were stored at −20 °C in 30% glycerol.

### 3.3. Determination of Activity of Transaminases

The activities of the purified transaminase variants were studied using the conversion of (*R*)-1-phenylethylamine resulting in the formation of acetophenone [51], which was quantified photometrically at 245 nm over time using the Infinite^®^ 200 PRO (TECAN) plate reader in UV-transparent microtiter plates (UV-Star, Greiner Bio-One GmbH, Berlin, Germany). The assay was performed with 2.5 mM (*R*)-1-phenylethylamine as amine donor and 2.5 mM pyruvate as amine acceptor in 1.25–2.5% DMSO, 50 mM Davies buffer pH 6.5–9.5 at 30 °C. One unit (U) activity was defined as the formation of 1 µmol acetophenone per minute. All measurements were performed in triplicates.

### 3.4. Docking Experiments

The geometrical optimizations of substrates were carried out with the *ChemBio3D Ultra 13.0* suite program using the MM2 basis set. Dockings were performed with the *AutoDock 4.2.6* suite [55]. All the water molecules were deleted in the docking simulation, and the grid box was centered on the carbon atom of the internal aldimine of the cofactor PLP. Other parameters were kept at the default settings. The resulting ligand and original protein data files were used to generate Figure 4 using the *PyMOL 2.3.0* program [49] (Table S5). The water molecule shown in Figure 4 comes from the crystal structure of ATA-Afu (PDB: 4CHI).

### 3.5. pKa Prediction and Evaluation

The p*K*a values of the residue H53 of ATA-Afu or its E49Q variant were predicted by the program *PropKa 3.3* (developed by the Jensen group) [52]. *PropKa 3.3* predicts the p*K*a values of ionizable groups in proteins based on their 3D structure. The 3D structure of ATA-Afu was downloaded from the PDB database (PDB code: 4CHI) and was used as a structure template to predict the 3D homology models of its E49Q variant, using the program YASARA based on its homology model generation algorithm [45]. The pdb file served as input for *PropKa 3.3* and the predicted p*K*a values of all the ionizable residues were calculated automatically.

The p*K*a value of the internal aldimine group of the cofactor of ATA-Afu-E49Q was measured using a literature method [42]. The absorption spectra of ATA-Afu-E49Q at pH 6.5–9.5 were determined using the Infinite^®^ 200 PRO (TECAN) plate reader in UV-transparent microtiter plates (UV-Star, Greiner Bio-One GmbH). These pH-dependent spectral changes have been known to reflect the ionization state of the imine nitrogen of the internal aldimine bond formed between K179 and PLP, and the protonated form of the internal aldimine of ATA-Afu-E49Q is thought to have an absorption band around 410 nm, which was titratable and resulted in a p*K*a value of 9.0 (Figure S3, Supplementary Materials).

### 3.6. Asymmetric Synthesis of (R)-1-Phenylethylamine

Asymmetric synthesis of (*R*)-PEA using purified ATA-Afu or its E49Q variant was done at pH 7.5 or 8.5, respectively. HPLC calibration curves are shown in Figure S4. To a reaction vial (20 mL) with a screwcap, the following components were added to give a final volume of 10 mL: sodium phosphate buffer (50 mM, pH 7.5 or 8.5) including PLP (0.1 mM); *rac*-alanine (100 mM); oxidized nicotinamide adenine dinucleotide (NAD^+^, 1 mM); glucose dehydrogenase (1 U mL^−1^); ᴅ-glucose (50 mM) and lactate dehydrogenase (3 U mL^−1^); 0.050 mL stock solution of acetophenone (100 mM in DMSO); purified protein (5 mg). The reaction vials were incubated at 30 °C, 200 rpm for 48 h. To monitor the progression of the reaction, samples (50 μL) were taken at different time points of the reaction (0, 2, 4, 6, 8, 10, 24 and 48 h), and mixed with acetonitrile (100 μL) and hydrochloric acid solution (100 mM, 50 μL) for HPLC analysis of the conversion of (*R*)-1-phenylethylamine. Concentrations of (*R*)-PEA and acetophenone were determined using an HPLC system with a Luna Omega 5 um Polar C18 (150 × 4.6 mm, Phenomenex), flow rate at 1 mL/min, detection at 210 nm, column temperature at 25 °C. The gradient elution started with H_2_O/CH_3_CN/TFA 90:10:0.1 (by volume) hold 3 min, ramped up to H_2_O/CH_3_CN/TFA 50:50:0.1 in 2 min, hold 1 min, then back to H_2_O/CH_3_CN/TFA 90:10:0.1 in 1 min, kept constant until the end of the run. Authentic standards were analyzed before the analysis of the reaction mixtures. Retention times: 3.87 min for (*R*)-1-PEA, 6.94 min for acetophenone.

## 4. Conclusions

In conclusion, we have identified key residues which affect the pH profiles of (*R*)-selective transaminases and demonstrated a shift of pH optima resulting in improved catalytic activity. The possible mechanism behind the different pH optima found for the ATA variants was explored and confirmed by activity tests of different variants, together with computational calculations such as molecular docking and p*K*a prediction. This revealed that the variant ATA-Afu-E49Q exhibited higher reaction rates in the synthesis of (*R*)-1-phenylethylamine at pH 7.5.

## Data Availability

Any data or material that support the findings of this study can be made available by the corresponding author upon request.

## Supplementary Materials

The following supporting information can be downloaded at: https://www.mdpi.com/article/10.3390/ijms232315347/s1.
