# Supplementary material for: Shifting the pH Optima of (R)-Selective Transaminases by Protein Engineering
Source: Int J Mol Sci. 2022 Dec 5;23(23):15347. doi: 10.3390/ijms232315347 (PMC9736275; doi:10.3390/ijms232315347)
Supplement: Supplementary file 1 [file ijms-23-15347-s001.zip › ijms-2043550-supplementary.pdf]

Supporting Information

Shifting the pH Optima of (*R*)-Selective Transaminases by Protein Engineering

Chao Xiang,[a,†] Yu-Fei Ao,[a,b,c,†] Matthias Höhne,[a] Uwe T. Bornscheuer[a,*]

[a] *Department of Biotechnology and Enzyme Catalysis, Institute of Biochemistry, University of Greifswald, Greifswald, 17487, Germany*

[b] *Beijing National Laboratory for Molecular Sciences, CAS Key Laboratory of Molecular Recognition and Function, Institute of Chemistry, Chinese Academy of Sciences, Beijing, 100190, China*

[c] *University of Chinese Academy of Sciences, Beijing 100049, China*

Email: uwe.bornscheuer@uni-greifswald.de

Table of Contents

Table S1. List of Primers	S2
Table S2. Detailed specific activity data	S3
Figure S1. Lineweaker-Burk linearization plots	S5
Table S3. Kinetic parameters of reactions	S6
Figure S2. pH activity profiles of mutants ATA-Gze-H53L and ATA-Ate-H55L	S7
Figure S3. pH-dependent spectral changes of the ATA-Afu-E49Q	S8
Figure S4. HPLC calibration curves of (*R*)-PEA	S9
Table S4. Detailed data of asymmetric synthesis	S10
Table S5. Docking results	S11
Figure S5. The superposed structure of substrate	S11
Figure S6. Evolutionary conservation alignment of transaminases	S12
Protein and DNA sequences of the enzymes studied	S13

Table S1. List of Primers.

Primer	Sequence (5' to 3')
ATA-Afu-E49Q Fw	CAGGGTTTTATGCATAGTGATCTGACC
ATA-Afu-E49Q Rv	ATCCAGCAGCGGGATGC
ATA-Afu-T123K Fw	AAAGGTGTTCGTGGTTCTAAACCGG
ATA-Afu-T123K Rv	CAGACCACGGGTAACAATAACTTCC
ATA-Afu-G127E Fw	ACCGGTGTTCGTGAATCTAAACCGGAAG
ATA-Afu-T123E Rv	CAGACCACGGGTAACAATAACTTCC
ATA-Gze-Q49E Fw	GAAGGCTTTATGCATGGTGATCTG
ATA-Gze-Q49E Rv	ATCCATCAGCGGAATGCG
ATA-Gze-K123T Fw	CCGGTTCGCGAAGCAAAACCGGGTG
ATA-Gze-K123T Rv	CGTCAGGCCACGCGTAACGATC
ATA-Gze-E127G Fw	CCGGTTCGCGGTGCAAAACCGGGTG
ATA-Gze-P127G Rv	TTTCAGGCCACGCGTAACGATC
ATA-Ate-Q51E Fw	GAAGGTTTTATGCATAGCGATC
ATA-Ate-Q51E Rv	ATCCAGCAGCGGAATAC
ATA-Ate-K125T Fw	ACCGGTGTTCGTGGCACCC
ATA-Ate-K125T Rv	CAGACCACGGGTAACAATCAG
ATA-Ate-G129E Fw	GAAACCCGTCCGGAAGATATCGTG
ATA-Ate-G129E Rv	ACGAACACCTTTCAGACCACGG

Table S2. Detailed specific activity data

Variants	pH, specific activity[a] [U mg^{-1}]							
	pH = 6	pH = 6.5	pH = 7	pH = 7.5	pH = 8	pH = 8.5	pH = 9	pH = 9.5
ATA-Afu	1.158 ± 0.010	2.247 ± 0.018	3.167 ± 0.042	3.588 ± 0.077	3.960 ± 0.128	4.032 ± 0.061	3.238 ± 0.044	2.240 ± 0.085
ATA-Afu-E49Q	0.736 ± 0.026	2.190 ± 0.039	4.827 ± 0.061	6.923 ± 0.228	6.078 ± 0.306	3.596 ± 0.016	2.199 ± 0.119	0.900 ± 0.030
ATA-Afu-T123K	1.644 ± 0.030	3.198 ± 0.025	4.784 ± 0.032	5.359 ± 0.025	5.553 ± 0.079	5.770 ± 0.131	4.584 ± 0.079	3.231 ± 0.094
ATA-Afu-G127E	0.472 ± 0.016	0.802 ± 0.017	1.133 ± 0.031	1.246 ± 0.039	1.274 ± 0.069	1.294 ± 0.024	1.097 ± 0.012	0.847 ± 0.025
ATA-Afu-E49Q/T123K	0.562 ± 0.027	1.508 ± 0.015	3.224 ± 0.109	4.468 ± 0.072	3.655 ± 0.163	2.788 ± 0.100	1.455 ± 0.114	0.692 ± 0.008
ATA-Gze	0.633 ± 0.026	2.406 ± 0.036	3.622 ± 0.009	2.663 ± 0.060	1.510 ± 0.033	0.976 ± 0.024	0.501 ± 0.020	0.287 ± 0.015
ATA-Gze-Q49E	0.279 ± 0.010	0.713 ± 0.002	1.305 ± 0.008	1.610 ± 0.008	1.638 ± 0.037	1.599 ± 0.008	1.295 ± 0.026	0.801 ± 0.016
ATA-Gze-E127G	0.458 ± 0.001	1.303 ± 0.018	2.780 ± 0.058	3.141 ± 0.036	1.655 ± 0.094	1.012 ± 0.019	0.332 ± 0.036	0.227 ± 0.013
ATA-Gze-K123T	0.852 ± 0.003	2.514 ± 0.173	4.630 ± 0.026	3.789 ± 0.185	1.831 ± 0.142	0.957 ± 0.036	0.482 ± 0.034	0.241 ± 0.004
ATA-Ate	0.196 ± 0.005	1.140 ± 0.013	2.540 ± 0.050	3.067 ± 0.021	2.273 ± 0.076	1.665 ± 0.013	0.856 ± 0.015	0.467 ± 0.031
ATA-Ate-Q51E	0.147 ± 0.026	0.548 ± 0.012	0.902 ± 0.007	0.967 ± 0.003	1.062 ± 0.083	1.273 ± 0.080	1.018 ± 0.015	0.821 ± 0.020
ATA-Ate-K125T	0.178 ± 0.008	1.088 ± 0.023	2.470 ± 0.020	2.827 ± 0.051	2.027 ± 0.079	1.567 ± 0.017	0.808 ± 0.011	0.425 ± 0.027
ATA-Ate-G129E	0.181 ± 0.006	1.002 ± 0.009	1.992 ± 0.012	2.173 ± 0.044	1.583 ± 0.033	1.077 ± 0.005	0.485 ± 0.012	0.332 ± 0.012

ATA-Ate-Q51E/K125T	0.185 ± 0.004	0.680 ± 0.001	1.131 ± 0.003	1.349 ± 0.016	1.353 ± 0.016	1.357 ± 0.015	1.156 ± 0.017	0.891 ± 0.014
ATA-Afu-H53L	0.023 ± 0.002	0.022 ± 0.001	0.022 ± 0.001	0.023 ± 0.002	0.018 ± 0.001	0.017 ± 0.001	0.015 ± 0.001	0.028 ± 0.001
ATA-Afu-H53F	0.011 ± 0.001	0.005 ± 0.003	0.006 ± 0.001	0.005 ± 0.001	0.007 ± 0.001	0.005 ± 0.003	0.001 ± 0.002	0.002 ± 0.001
ATA-Afu-E49Q/H53L	0.237 ± 0.007	0.336 ± 0.015	0.363 ± 0.017	0.367 ± 0.005	0.374 ± 0.006	0.406 ± 0.029	0.373 ± 0.016	0.334 ± 0.005
ATA-Gze-H53L	0.078 ± 0.001	0.148 ± 0.002	0.181 ± 0.002	0.305 ± 0.002	0.282 ± 0.007	0.184 ± 0.003	0.119 ± 0.003	0.070 ± 0.001
ATA-Ate-H55L	0.112 ± 0.003	0.334 ± 0.007	0.376 ± 0.002	0.379 ± 0.001	0.390 ± 0.004	0.384 ± 0.003	0.372 ± 0.001	0.337 ± 0.013

[a]Initial activity measured using the acetophenone assay (for (*R*)-PEA and pyruvate). One unit (U) was defined as the formation of 1 µmol acetophenone per minute. All measurements were performed in triplicates.

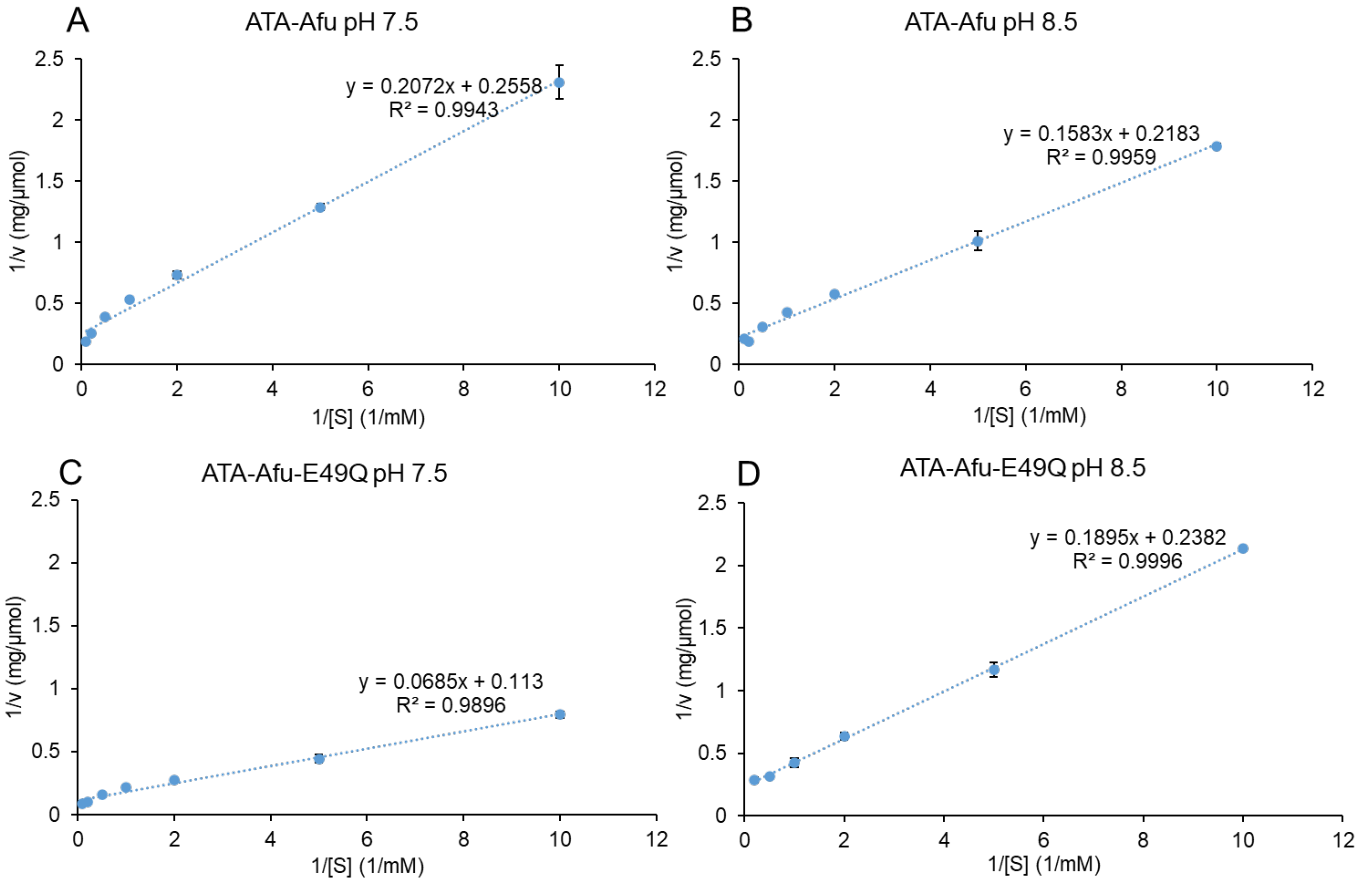

Figure S1. Lineweaker-Burk linearization plots of ATA-Afu and ATA-Afu-E49Q at pH 7.5 and 8.5. The kinetic parameters k_{cat} and K_m were calculated accordingly. All measurements were performed in triplicates.

Table S3. Kinetic parameters of the reactions catalyzed by ATA-Afu and ATA-Afu-E49Q

pH	Enzyme	K_m [mM]	k_{cat} [s^{-1}]	k_{cat}/K_m [mM s^{-1}]
7.5	ATA-Afu	0.81 ± 0.07	4.91 ± 0.19	6.06
8.5	ATA-Afu	0.73 ± 0.02	5.77 ± 0.13	7.90
7.5	ATA-Afu-E49Q	0.61 ± 0.04	11.11 ± 0.43	18.21
8.5	ATA-Afu-E49Q	0.80 ± 0.03	5.27 ± 0.19	6.59

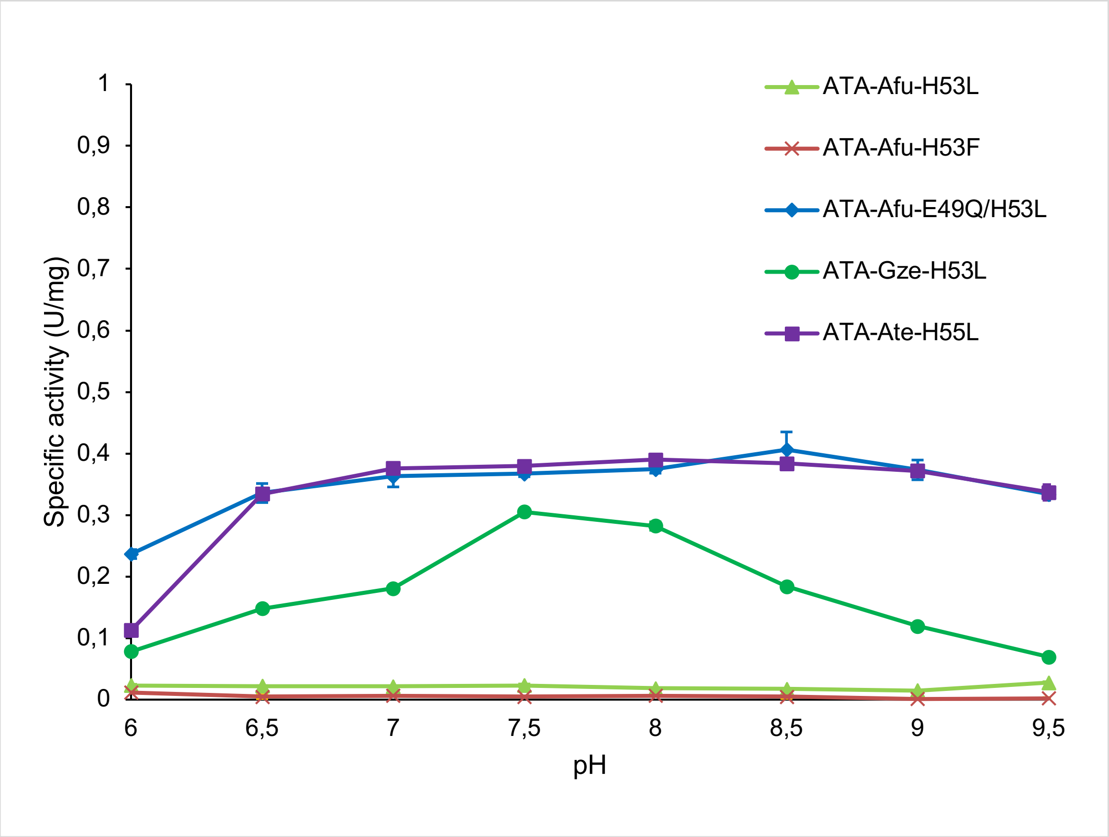

Figure S2. pH activity profiles of mutants ATA-Gze-H53L and ATA-Ate-H55L. Data points corresponded to the mean values of three independent experiments.

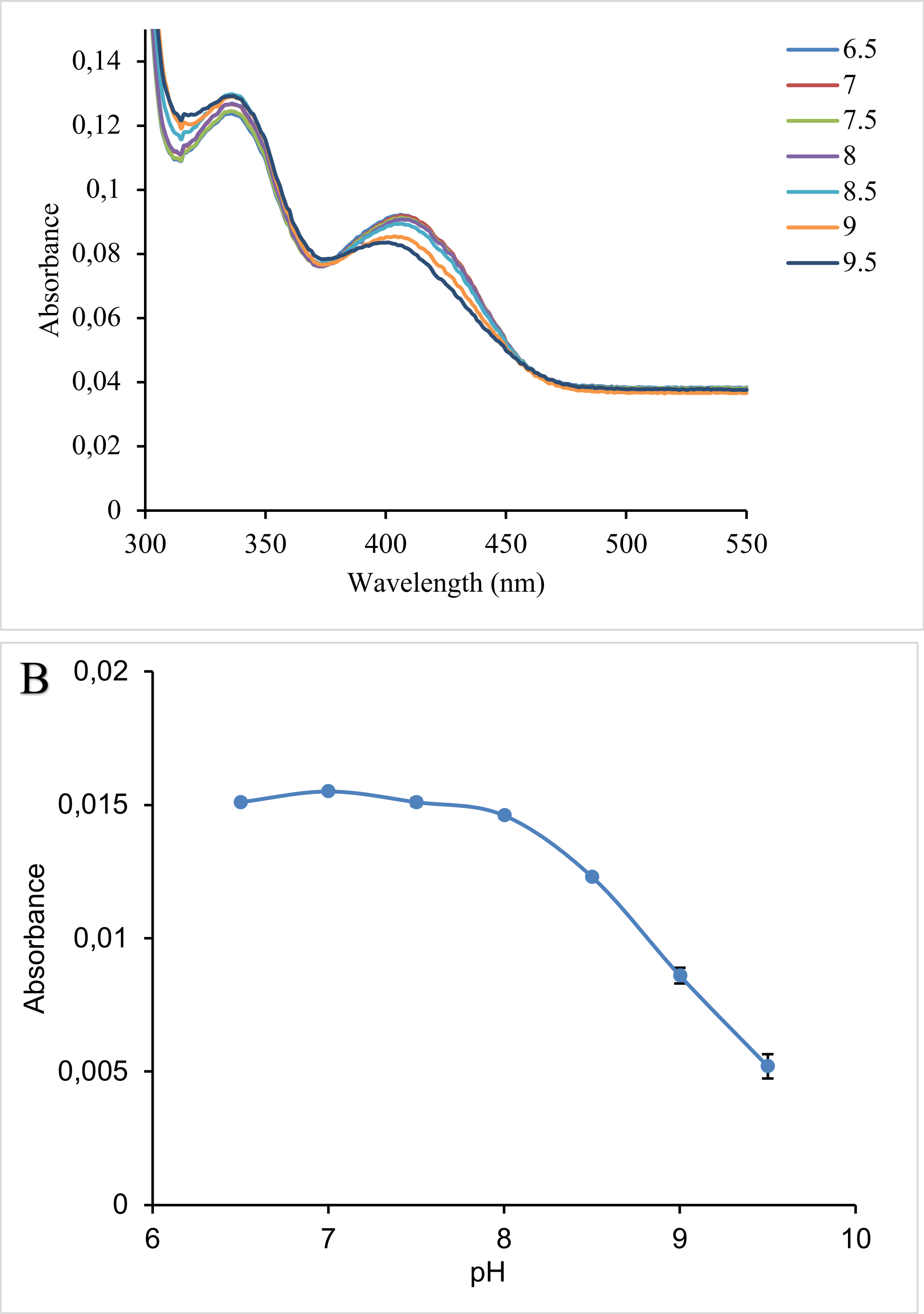

Figure S3. pH-dependent spectral changes of the PLP form of ATA-Afu-E49Q. (A) Absorption spectra of ATA-Afu-E49Q in the pH range from 6.5 to 9.5. (B) Absorption changes at 410 nm as a function of the pH. ATA-Afu-E49Q was less stable at pH > 9.5, and thus the data could not be obtained at more alkaline pH values. All measurements were performed in triplicates.

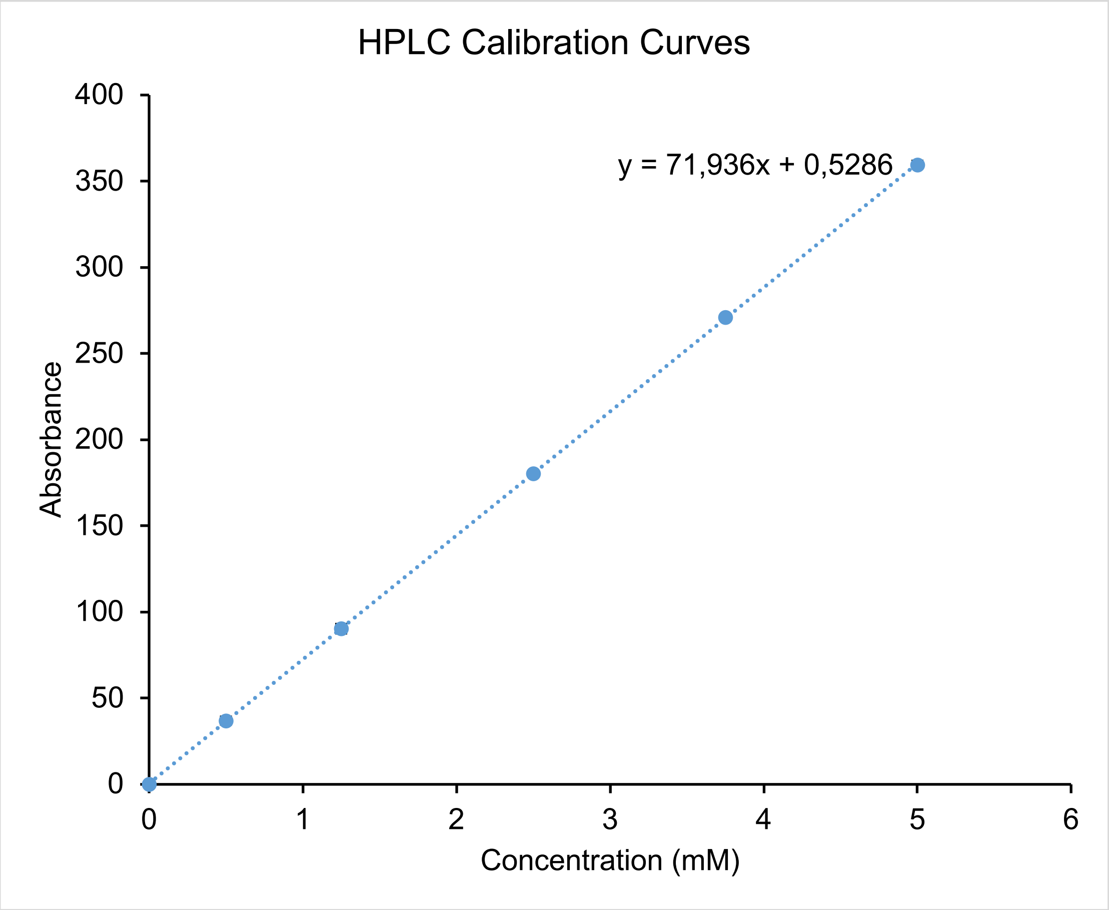

Figure S4. HPLC calibration curves of (*R*)-PEA. All measurements were performed in triplicates.

Table S4. Detailed data of asymmetric synthesis

pH	Enzyme	Conversion rates (%)							
		0 h	2 h	4 h	6 h	8 h	10 h	24 h	48 h
7.5	ATA-Afu	0 ± 0	15.56 ± 1.59	28.52 ± 1.83	37.96 ± 1.54	46.30 ± 2.40	53.61 ± 1.26	86.48 ± 2.65	98.06 ± 2.30
8.5	ATA-Afu	0 ± 0	20.56 ± 1.59	33.33 ± 1.64	42.22 ± 1.42	50.09 ± 1.73	55.93 ± 1.61	88.98 ± 2.87	98.06 ± 1.49
7.5	ATA-Afu-E49Q	0 ± 0	31.02 ± 1.67	54.44 ± 1.04	64.35 ± 2.45	71.30 ± 3.06	76.11 ± 2.62	98.33 ± 0.60	98.98 ± 0.26
8.5	ATA-Afu-E49Q	0 ± 0	21.94 ± 0.45	39.44 ± 0.68	50.93 ± 0.35	57.96 ± 1.82	63.70 ± 1.54	95.83 ± 1.86	99.07 ± 1.12

Assay conditions for asymmetric synthesis: 10 mL final volume, 50 mM phosphate buffer at pH 7.5 or 8.5, 0.1 mM PLP, 5 mM ketone, 100 mM D,L-alanine, NAD^+ (1 mM), GDH (1 U ml^{-1}), D-glucose (50 mM) and LDH (3 U ml^{-1}), DMSO 5%, 5 mg purified enzyme, 30 °C under stirring. All measurements were performed in triplicates.

Table S5. Docking results.

Rank	Binding energy (kcal/mol)
1	-5.21
2	-4.51
3	-4.46

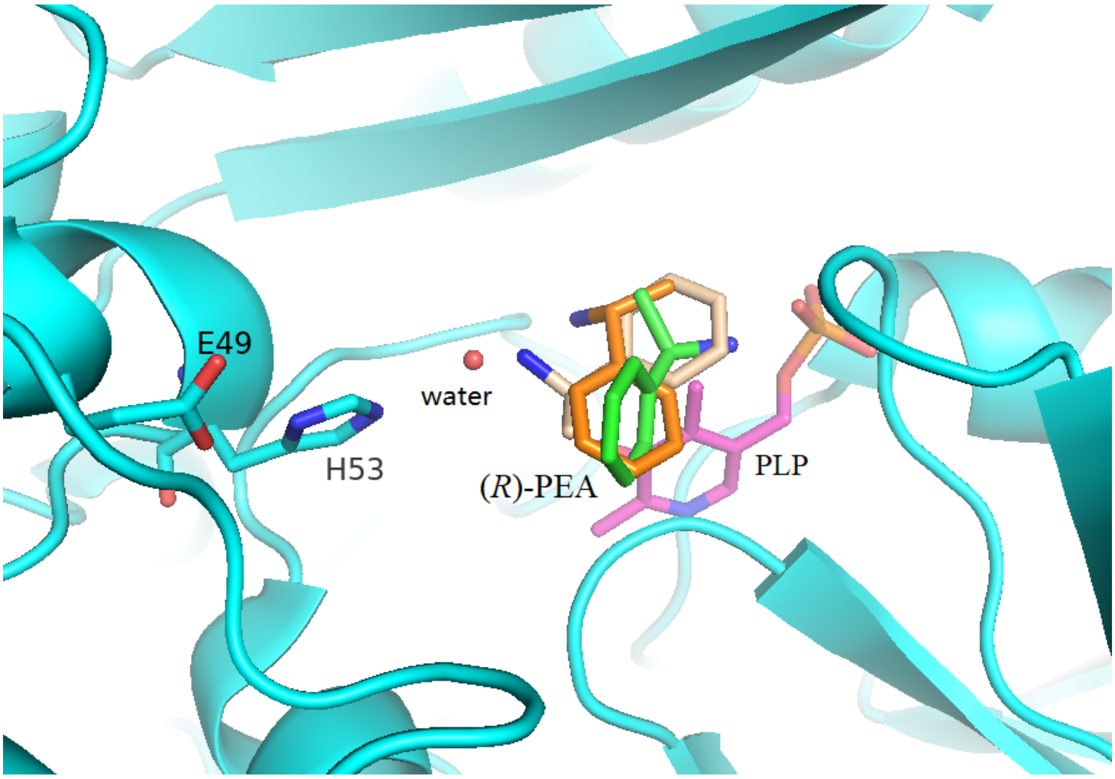

Figure S5. The superposed structure of substrate (*R*)-PEA accommodated in the active site of ATA-Afu. The protein is shown in cyan. The carbon atoms of the three docking results of substrate are colored orange, wheat and green, respectively. The figure was created using PyMOL.

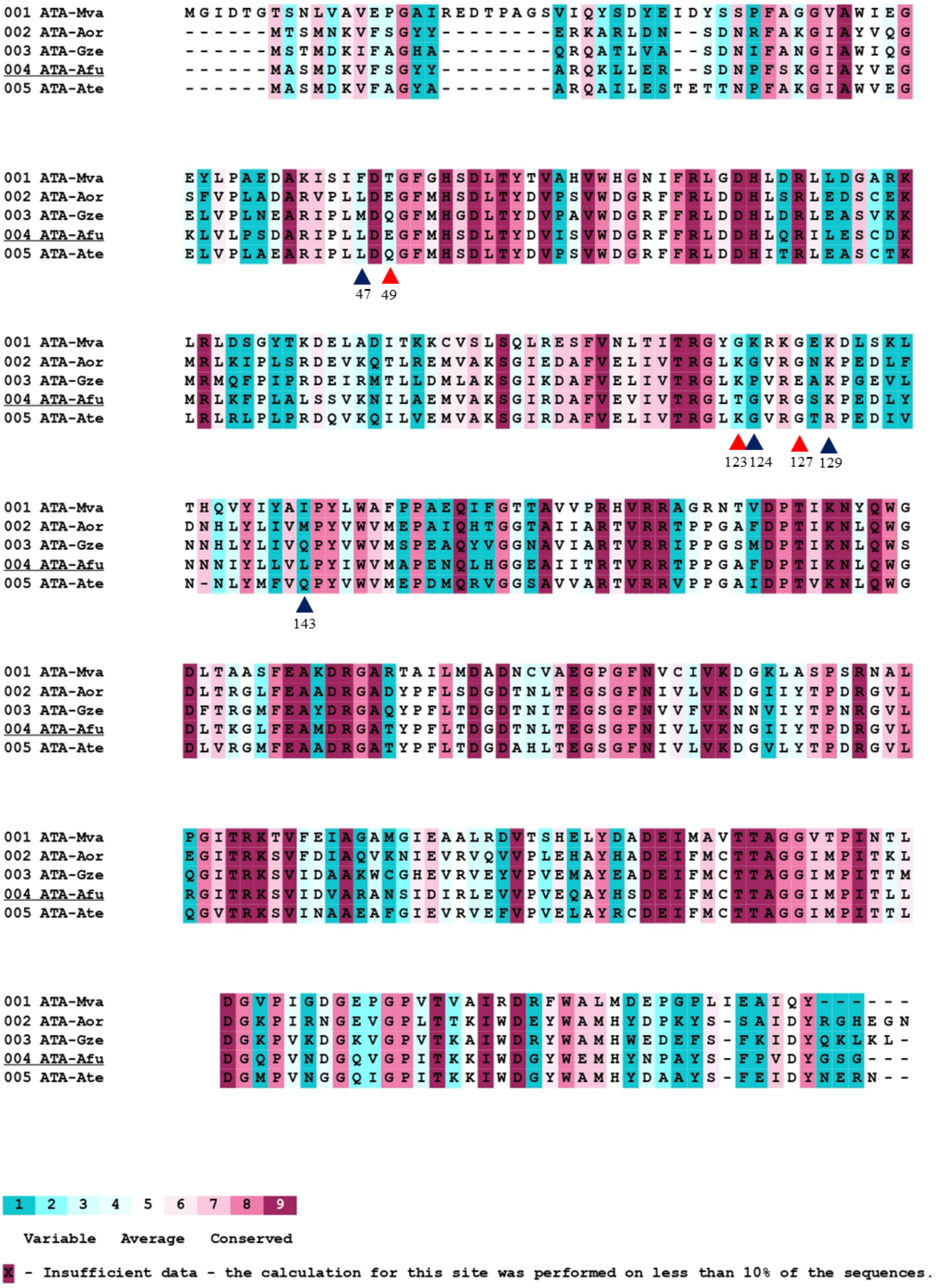

Figure S6. Evolutionary conservation alignment of transaminases. The residues in the substrates tunnel area are marked with triangles. Among them, E49, T123 and G127 present in ATA-Afu (red colored triangles) have differently charged side chains compared to the other two ATAs. The alignment tool needs at least five homologous proteins, therefore two homologous transaminases ATA-Mva (Uniprot: Mvan_4516) and ATA-Aor (Uniprot: CNMCM6457_008109) were added to the alignment. The figure was created using the online alignment tools *ConSurf* [46].

Protein and DNA sequences of the enzymes studied

>Protein sequence of ATA-Afu wildtype (Uniprot: AFUA_7G06900):

MASMDKVFSGYYARQKLLERSDNPFSKGIAYVEGKLVLPSDARIPLLDEGFMHSDLT
YDVISVWDGRFFRLDDHLQRILESCDKMRLKFPLALSSVKNILAEMVAKSGIRDAFVE
VIVTRGLTGVRGSKPEDLYNNNIYLLVLPYIWVMAPENQLHGGEAIITRTVRRTPPGA
FDPTIKNLQWGDLTKGLFEAMDRGATYPFLTDGDTNLTEGSGFNIVLVKNGIIYTPDR
GVLRGITRKSVIDVARANSIDIRLEVVPVEQAYHSDEIFMCTTAGGIMPITLLDGQPVN
DGQVGPITKKIWDGYWEMHYNPAYSFPVDYGSGSGSHHHHHH

>DNA sequence of ATA-Afu wildtype (Uniprot: AFUA_7G06900)

ATGGCATCTATGGATAAAGTTTTTAGTGGTTACTACGCGCGCCAGAAACTGCTGG
AACGTAGTGATAATCCGTTCAGCAAAGGTATTGCCTATGTTGAAGGCAAACTGGT
GCTGCCGAGTGATGCACGCATCCCGCTGCTGGATGAAGGTTTTATGCATAGTGAT
CTGACCTACGATGTTATTAGCGTGTGGGATGGCCGTTTCTTTCGCCTGGATGATCA
CCTGCAGCGCATCCTGGAAAGCTGCGATAAAATGCGTCTGAAATTTCCGCTGGCA
CTGAGCTCTGTTAAAAACATTCTGGCAGAAATGGTGGCGAAAAGCGGCATTCGTG
ATGCGTTCGTGGAAGTTATTGTTACCCGTGGTCTGACCGGTGTTCGTGGTTCTAAA
CCGGAAGATCTGTATAACAATAACATTTACCTGCTGGTTCTGCCGTATATCTGGGT
GATGGCGCCGGAAAATCAGCTGCATGGCGGTGAAGCCATTATTACCCGTACCGTT
CGTCGCACCCCGCCGGGTGCATTTGATCCGACCATTAAAAACCTGCAGTGGGGTG
ATCTGACCAAAGGCCTGTTTGAAGCCATGGATCGTGGTGCAACCTATCCGTTCCT
GACGGATGGCGATACCAATCTGACGGAAGGCTCTGGTTTCAATATCGTTCTGGTG
AAAAACGGCATTATCTACACCCCGGATCGTGGTGTTCTGCGCGGCATTACGCGTA
AATCTGTTATCGATGTGGCGCGCGCCAACAGTATTGATATCCGTCTGGAAGTGGT
TCCGGTGGAACAGGCGTATCATAGCGATGAAATTTTTATGTGTACCACGGCCGGC
GGTATTATGCCGATCACCCTGCTGGATGGTCAGCCGGTTAATGATGGTCAAGTGG
GCCCGATTACCAAGAAAATTTGGGATGGCTATTGGGAAATGCACTATAACCCGGC
GTACAGCTTCCCGGTGGATTACGGCTCTGGTTCAGGATCCCATCATCATCATCATC
ATTGA

>Protein sequence of ATA-Gze wildtype (Uniprot: FG01766.1)

MSTMDKIFAGHAQRQATLVASDNIFANGIAWIQGELVPLNEARIPLMDQGFMHGDLT
YDVPAVWDGRFFRLDDHLDRLEASVKKMRMQFPIPRDEIRMTLLDMLAKSGIKDAF
VELIVTRGLKPVREAKPGEVLNNHLYLIVQPYVWVMSPEAQYVGGNAVIARTVRRIP
PGSMDPTIKNLQWSDFTRGMFEAYDRGAQYPFLTDGDTNITEGSGFNVVFVKNNVIY
TPNRGVLQGITRKSVIDAAKWCGHEVRVEYVPVEMAYEADEIFMCTTAGGIMPITTM
DGKPVKDGKVGPVTKAIWDRYWAMHWEDEFSFKIDYQKLKLSGSHHHHHH

>DNA sequence of ATA-Gze wildtype (Uniprot: FG01766.1)

ATGAGTACCATGGATAAAATTTTTGCAGGCCATGCGCAGCGTCAGGCAACGCTGG
TTGCGAGCGATAACATCTTCGCCAATGGCATTGCATGGATCCAGGGTGAACTGGT
GCCGCTGAACGAAGCGCGCATTCCGCTGATGGATCAGGGCTTTATGCATGGTGAT
CTGACCTATGATGTGCCGGCGGTTTGGGATGGCCGTTTCTTTCGCCTGGATGATCA
CCTGGATCGTCTGGAAGCCTCTGTGAAGAAAATGCGCATGCAGTTTCCGATTCCG
CGTGATGAAATCCGCATGACCCTGCTGGATATGCTGGCAAAAAGTGGTATTAAAG
ATGCGTTCGTGGAACTGATCGTTACGCGTGGCCTGAAACCGGTTCGCGAAGCAAA
ACCGGGTGAAGTGCTGAACAATCACCTGTATCTGATTGTGCAGCCGTACGTGTGG
GTTATGAGCCCGGAAGCCCAGTATGTGGGCGGTAACGCCGTTATTGCGCGCACCG
TTCGTCGCATCCCGCCGGGTAGCATGGATCCGACCATCAAAAATCTGCAGTGGTC
TGATTTTACGCGTGGCATGTTCGAAGCGTATGATCGCGGTGCCCAGTACCCGTTTC
TGACCGATGGTGATACCAACATTACGGAAGGCAGCGGTTTCAACGTGGTTTTCGT
GAAAAACAACGTTATCTACACCCCGAATCGTGGCGTTCTGCAGGGTATTACGCGC
AAATCTGTGATCGATGCGGCCAAATGGTGCGGCCATGAAGTGCGTGTTGAATATG
TGCCGGTTGAAATGGCGTACGAAGCCGATGAAATTTTCATGTGTACCACGGCGGG
CGGTATTATGCCGATCACCACGATGGATGGTAAACCGGTGAAAGATGGCAAAGT
GGGTCCGGTTACCAAAGCCATTTGGGATCGCTATTGGGCAATGCACTGGGAAGAT
GAATTTAGCTTCAAAATCGATTACCAGAAACTGAAACTGTCAGGATCCCATCATC
ATCATCATCATTGA

>Protein sequence of ATA-Ate wildtype (Uniprot: ATEG_10023)

MASMDKVFAGYAARQAILESTETTNPFAKGIAWVEGELVPLAEARIPLLDQGFMHSD
LTYDVPSVWDGRFFRLDDHITRLEASCTKLRLRLPLPRDQVKQILVEMVAKSGIRDAF
VELIVTRGLKGVRGTRPEDIVNNLYMFVQPYVWVMEPDMQRVGGSAVVARTVRRV
PPGAIDPTVKNLQWGDLVRGMFEAADRGATYPFLTDGDAHLTEGSGFNIVLVKDGV
LYTPDRGVLQGVTRKSVINAAEAFGIEVRVEFVPVELAYRCDEIFMCTTAGGIMPITT
LDGMPVNGGQIGPITKKIWDGYWAMHYDAAYSFEIDYNERNSGSHHHHHH

>DNA sequence of ATA-Ate wildtype (Uniprot: ATEG_10023)

ATGGCATCAATGGATAAGGTTTTTGCCGGTTATGCAGCACGTCAGGCAATTCTGGA
AAGCACCGAAACCACCAATCCGTTTGCAAAAGGTATTGCATGGGTTGAAGGTGAA
CTGGTTCCGCTGGCAGAAGCACGTATTCCGCTGCTGGATCAGGGTTTTATGCATAG
CGATCTGACCTATGATGTTCCGAGCGTTTGGGATGGTCGTTTTTTTCGTCTGGATGA
TCATATTACCCGTCTGGAAGCCAGCTGTACCAAACTGCGTCTGCGTCTGCCGCTGC
CTCGTGATCAGGTTAAACAAATTCTGGTTGAAATGGTTGCCAAAAGCGGTATTCGT
GATGCATTTGTGGAACTGATTGTTACCCGTGGTCTGAAAGGTGTTCGTGGCACCCG
TCCGGAAGATATCGTGAATAATCTGTATATGTTTGTGCAGCCGTATGTTTGGGTTATG
GAACCGGATATGCAGCGTGTTGGTGGTAGCGCAGTTGTTGCACGTACCGTTCGTCG
TGTTCCGCCTGGTGCAATTGATCCGACCGTTAAAAATCTGCAGTGGGGTGATCTGG
TTCGTGGTATGTTTGAAGCAGCAGATCGTGGTGCAACCTATCCGTTTCTGACCGAT
GGTGATGCACATCTGACCGAAGGTAGCGGTTTTAACATTGTGCTGGTGAAAGATGG
TGTTCTGTATACACCGGATCGTGGTGTTCTGCAGGGTGTTACACGTAAAAGCGTGA
TTAATGCAGCAGAAGCCTTTGGTATTGAAGTGCGTGTTGAATTTGTTCCGGTTGAA
CTGGCATATCGCTGTGATGAAATTTTTATGTGTACCACCGCAGGCGGTATTATGCCG
ATTACCACCCTGGATGGTATGCCGGTTAATGGTGGTCAGATTGGTCCGATTACCAAA
AAAATTTGGGATGGCTATTGGGCAATGCATTATGATGCAGCCTATAGCTTTGAAATT
GATTATAATGAACGCAATTCAGGATCCCATCATCATCATCATCATTGA
